# A Smartphone-Based Technique to Detect Dynamic User Preferences for Tailoring Behavioral Interventions: Observational Utility Study of Ecological Daily Needs Assessment

**DOI:** 10.2196/18609

**Published:** 2020-11-13

**Authors:** Ginger E Nicol, Amanda R Ricchio, Christopher L Metts, Michael D Yingling, Alex T Ramsey, Julia A Schweiger, J Philip Miller, Eric J Lenze

**Affiliations:** 1 Healthy Mind Lab Department of Psychiatry Washington University School of Medicine St. Louis, MO United States; 2 Department of Pathology and Laboratory Medicine College of Medicine Medical University of South Carolina Charleston, SC United States; 3 Department of Psychiatry Washington University School of Medicine St. Louis, MO United States; 4 Division of Biostatistics Washington University School of Medicine St. Louis, MO United States

**Keywords:** mobile health, telemedicine, needs assessment, healthy lifestyle, ecological momentary assessment, mobile applications, behavior intervention, behavior therapy

## Abstract

**Background:**

Mobile health apps are promising vehicles for delivering scalable health behavior change interventions to populations that are otherwise difficult to reach and engage, such as young adults with psychiatric conditions. To improve uptake and sustain consumer engagement, mobile health interventions need to be responsive to individuals’ needs and preferences, which may change over time. We previously created an ecological daily needs assessment to capture microprocesses influencing user needs and preferences for mobile health treatment adaptation.

**Objective:**

The objective of our study was to test the utility of a needs assessment anchored within a mobile app to capture individualized, contextually relevant user needs and preferences within the framework of a weight management mobile health app.

**Methods:**

Participants with an iOS device could download the study app via the study website or links from social media. In this fully remote study, we screened, obtained informed consent from, and enrolled participants through the mobile app. The mobile health framework included daily health goal setting and self-monitoring, with up to 6 daily prompts to determine in-the-moment needs and preferences for mobile health–assisted health behavior change.

**Results:**

A total of 24 participants downloaded the app and provided e-consent (22 female; 2 male), with 23 participants responding to at least one prompt over 2 weeks. The mean length of engagement was 5.6 (SD 4.7) days, with a mean of 2.8 (1.1) responses per day. We observed individually dynamic needs and preferences, illustrating daily variability within and between individuals. Qualitative feedback indicated preferences for self-adapting features, simplified self-monitoring, and the ability to personalize app-generated message timing and content.

**Conclusions:**

The technique provided an individually dynamic and contextually relevant alternative and complement to traditional needs assessment for assessing individually dynamic user needs and preferences during treatment development or adaptation. The results of this utility study suggest the importance of personalization and learning algorithms for sustaining app engagement in young adults with psychiatric conditions. Further study in broader user populations is needed.

## Introduction

### Background

Digital health interventions are being developed at a rapid rate, particularly for behavioral management of chronic diseases like diabetes, heart disease, and stroke, which account for greater than 90% of health care expenditures and more than 75% of deaths in the United States [1,2]. Such interventions promise increased access and scalability across health care systems. Mobile health (mHealth) interventions in particular offer immediate engagement, often titratable to user preferences, with the ability to deploy assessments that incorporate both self-report and sensor data. However, approximately half of users disable health apps within 2 weeks of download, with loss of interest a commonly cited reason for app disablement [3]. Barriers to and facilitators of engagement, adherence, and motivation all likely vary over time, impacted by unique, context-dependent needs and preferences both intrinsic and extrinsic to the individual—variables that cannot be adequately assessed by traditional needs assessment methods. Person-centered strategies to optimize patient engagement are critically needed for mHealth apps to impart intended benefits, and for mHealth interventions to become a mainstream way of delivering health care [4].

Obesity treatment is a case where eliciting patient preferences in treatment development is particularly important, as (1) patient engagement is a critical indicator of treatment success, (2) there is a range of treatment options, and (3) there are limited data to guide personalization of a treatment approach [5]. Only 20% to 30% of individuals seeking first-line behavioral weight loss treatment achieve clinically meaningful or sustained weight loss [6,7]. Consistent predictors of treatment response across weight loss studies include treatment engagement (eg, session attendance, homework completion) [8], adherence to self-monitoring activities (eg, food and activity logging, tracking weight) [9], and intrinsic motivation for health behavior change [10]. However, individuals with psychiatric conditions may experience unique and persistent barriers to engagement in healthy eating and exercise [11,12]. For example, parents of adolescents with psychiatric illness who participated in a family-based weight loss intervention reported that major barriers to participation were pragmatic (transportation to and from in-person visits), or were directly related to the psychiatric condition (shame and avoidance). These individuals expressed preferences for digital self-monitoring and support delivered via a mobile device over in-person treatment. Moreover, participants indicated that needs and barriers varied from day to day, and that an intervention adaptive to their dynamically changing needs was important [13]. As the availability of mobile devices among mentally ill youth increases [14], this mode of treatment becomes a feasible way to increase access and engagement [15]. However, to our knowledge, no mHealth approaches to health promotion, including weight management, have been adapted to engage patients with early-onset psychiatric conditions, where prevention is a primary public health concern [16,17].

Traditional behavioral treatment adaptation approaches use a range of methods (eg, semistructured interviews, checklists, self-report questionnaires, focus groups) to elicit needs and preferences from potential recipients to inform treatment development and adaptation [18-20]. However, these methods are often employed at static assessment times, missing the dynamic effects of time and context on user needs and preferences, which are critical for treatment engagement. Digital data collection methods, such as ecological momentary assessment [21] and passively collected sensor data, can inform adaptation efforts. For example, just-in-time adaptive interventions track the dynamic effects of time and context on individual characteristics or behaviors and adapt, ideally in real time, based on a concrete measurable construct such as self-reported mood [22] or sedentary behavior [23]. With enough measurement time points, such ideographic assessment methods can be used to model and predict dynamic changes in symptom presentation or treatment response in a single individual, without the need for reference to a larger group [24,25]. However, few just-in-time adaptive interventions have collected or incorporated dynamic user preferences and perspectives into adaptation algorithms [26,27], and little is known about what types of data inputs are most relevant in building adaptive treatments for lifestyle change [28].

### Objective

To begin the process of adapting an existing interactive obesity treatment approach [29,30] for use in young people with psychiatric conditions, we created an ecological daily needs assessment. The intention was to capture dynamic user needs and preferences data as part of the adaptation process for developing a self-adapting treatment algorithm. However, the amount of lived-experience feedback needed to inform relevant treatment adaptation and, more importantly, the threshold for assessment fatigue, is not well understood [31]. The primary aim of this study was to evaluate the utility of the tool over a period of 2 weeks as part of an overall mHealth treatment adaptation effort, specifically to determine whether it could detect dynamic user needs and preferences. Secondarily, we aimed to determine the threshold for response fatigue in young adults with psychiatric conditions.

## Methods

### Participant Recruitment and Study Orientation

In this fully remote observational utility study, we recruited participants nationally via social media (Twitter, Facebook, and Instagram), as well as traditional methods, using an online research registry (ResearchMatch, Vanderbilt University); US national email listservs for college students, medical students, and residents; and word-of-mouth or flyers. These recruitment methods directed potential participants to the study website in order to download the free app. Once participants had downloaded the app, they were asked to complete a brief screening questionnaire, which included questions about comfort with and ability to use their device for answering preferences questions. Inclusion criteria were age between 18 and 45 years; access to a web-enabled device with the iPhone operating system (iOS; Apple Inc); ability to keep their device with them for most of the day over the following 2 weeks for the purpose of answering needs assessment prompts; and a history of a diagnosis of a psychiatric or psychological disorder. Since the app involved setting health goals for weight loss, a history of an eating disorder was exclusionary.

After reading about the study and passing the eligibility screen, participants signed consent on their device touch screen and received an email with the full signed consent document, which included email addresses and phone numbers for the study coordinator and principal investigator. Participants were asked about their preferred times for the following questions: (1) a time every morning to set a health intention for the day, (2) times the user typically ate breakfast, lunch, and dinner, or would be most likely to engage in physical activity, and (3) a time in the evening to answer questions about app usability and acceptability. Participants were then prompted 6 times daily each for eating and exercise needs assessments during their preferred times.

### Study Approval

This study was reviewed and approved by the Washington University Institutional Review Board and the Washington University Office of Information Security in December 2016. Following completion of development in August 2017, study enrollment was open from September 2017 to March 2018.

### Mobile App Development

As the first step in a larger, ongoing research program to adapt and test the effectiveness of an existing weight management intervention [29,30] for use in young adults with severe mental illness, we created a needs assessment tool for the purpose of conducting a digital needs assessment within the mHealth context. The theoretical behavior change framework underlying the overall program is based on increasing self-efficacy [32] for mental and physical health, by using mobile technology to reduce extraneous cognitive load associated with making health behavior changes [33] and to increase uptake of information and intervention engagement. The ecological needs assessment was embedded within a basic mHealth goal-setting framework.

### Needs Assessment Description

We developed the needs assessment using Status/Post, a digital platform (developed by CLM) that integrates the Apple ResearchKit (Apple Inc) framework with the REDcap (REDCap Consortium) web app through an application programming interface [34]. The app framework included a daily prompt for health intention setting and scaling, 6 prompts for needs assessment during the day designated by users as times they would be most likely to eat (3 prompts) or exercise (3 prompts), and reflection on goal progress at the end of the day (screen shots in Multimedia Appendix 1). The final survey each day consisted of feasibility, acceptability, and appropriateness questions adapted from Lyon and colleague’s contextualized technology adaptation process [35]. Feasibility questions focused on eliciting feedback from the user about the frequency and timing of question prompts, as well as messaging content. We conducted semistructured interviews with participants who had high (eg, responses on >80% of study days) and low (eg, responses on <25% of study days) engagement following completion of the 2-week utility study.

### Statistical Analysis

The recommended number of users needed for maximal detection of usability problems is 5 [36], and up to 25 users for comparative studies [37]. Anticipating a 50% attrition rate [3] over the 2-week study period, we enrolled 35 participants with the goal of at least 10 completers. We generated descriptive statistics (mean, frequencies, and proportions) for survey responses. We converted Likert-scale items from severity to a numeric rating (eg, 1 = not at all; 3 = very much). Over the course of the study, there were 235 potential eating prompt responses, comprised of 9 possible response options (ie, “Are you planning to eat? yes/no;” “Are you eating out? yes/no;” needs assessment question with 6 response options; and a free-text option for additional feedback), and 234 possible exercise prompt responses, comprising 8 possible response options (“Are you planning to exercise? yes/no;” needs assessment question with 6 response options; and free-text option for additional feedback). All participants with at least one postbaseline response were included in the analysis. We report responses to each survey question as number (n) and percentage of participants with a response in each category for a given question. We cleaned and analyzed data using the R software package version 3.1.1 2014-07-11, R.app 1.65 (R Foundation for Statistical Computing). We used all available data from all participants.

## Results

### Participant Characteristics

A total of 35 individuals downloaded the app, 24 consented to participate, and 23 proceeded past the orientation questions to answer at least one needs assessment question; 2 of them were male. Participants accessed the app via the study website (n=4), the online study registry (n=6), social media (n=6), an email listserv (n=6), or a flyer or by word-of-mouth (n=9). Participants were queried 6 times daily: 3 times daily during prespecified 1-hour periods when they were most likely to eat meals, and 3 times daily when they were most likely to exercise. Of the 23 participants who provided responses, 18 responded “Yes” to at least one eating query during the study, prompting the healthy eating needs assessment questions to be deployed 122 times over the study period. A total of 11 participants responded “Yes” to at least one exercise query during the study, prompting healthy activity needs assessment questions to be deployed 28 times during the study period.

### App Use and Engagement

**Figure 1:**
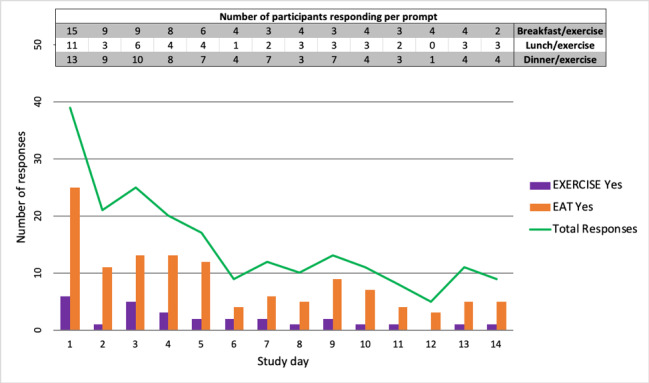
Overall study engagement shown by the number of total responses per day (green line), and “yes” responses per day for eating (orange bars) and exercise (purple bars). The table presents raw numbers of responses per day based on mealtime.

**Figure 2:**
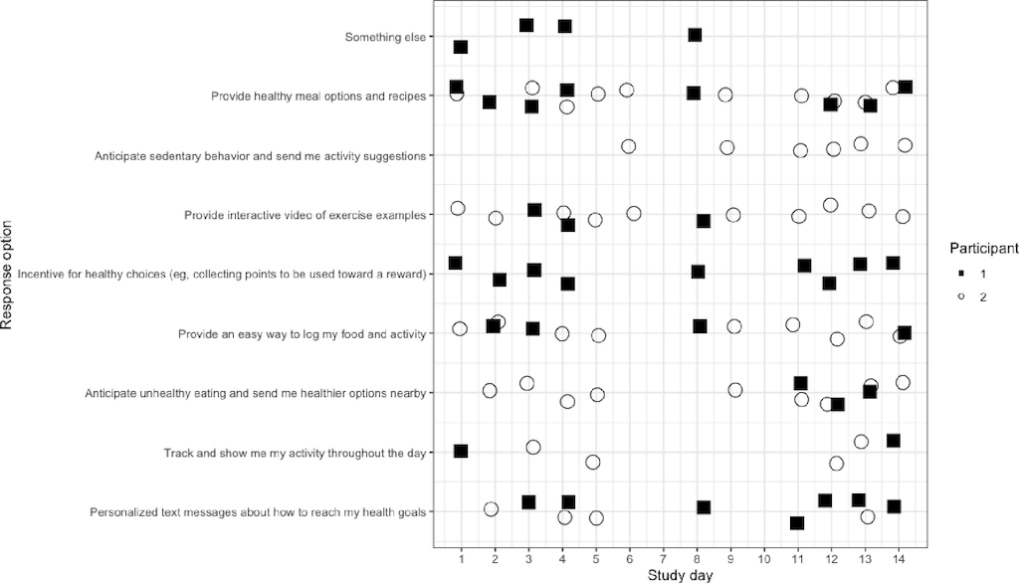
Responses on dynamic needs and preferences for healthy eating from 2 study participants over the 2-week study period.

The mean length of participation in the study was 5.6 (SD 4.7) days, with mean of 2.8 (1.1) responses per day. The mean number of responses per participant was 6.7 (3.0) over the 2-week study period. The earliest time to termination was 1 day; 2 individuals completed the entire 2 weeks. As Figure 1 shows, app use, defined as the number of participants responding to prompts, decreased by 46% (18/39) during the first day, with a varied but continual decrease in the number of respondents over the course of the study. However, the pooled daily response rate decreased to 80% (31/39) on day 1 and then remained stable at 67% (8/12) on day 7 through day 14 (6/9). Response rates remained relatively stable during the second week of the study, although individual responses varied across study day and assessment time point (Figure 2).

### User Needs and Preferences

As Table 1 shows, the top preferences for app functionality were receiving a reminder of the daily health goal, simplified self-monitoring for food intake, healthy eating and activity options nearby, and being able to see or track progress on goals throughout the day. More nuanced qualitative responses (eg, participants chose “something else” and entered a text response) suggested that participants wanted pragmatic assistance with making healthy eating selections (“Access to healthy preferred options or premade healthy options;” “An inventory of food in my house so I can figure out healthy options;” “Let me list at the beginning of the week what I have on hand and use those as suggestions”). Participants also indicated their preference for the app to learn their patterns and behaviors to anticipate their needs, as well as respond with personalized support (“Motivational messaging and a dashboard of my progress;” “Suggestions on healthy options in my area;” “Give me a list of 3-5 quick and healthy meal options;” “Someone to go with me and hold me accountable;” “Support me by checking in with me like a friend would”).

Responses to preference questions also revealed dynamic responses within and between individuals throughout each day over the course of the study (Figure 2).

**Table 1:** Pooled user needs and preferences for healthy eating and activity.

Preferences			Total responses selected, n (%)

**Eating preferences (n=122 questions deployed)**			
	Remind me of my daily health intention	51 (41.8)	
	Quick and easy option to log my food	50 (41.0)	
	Provide healthy eating options nearby	39 (32.0)	
	Show me my calorie intake for the day	37 (30.3)	
	Something else^a^	17 (13.9)	
	Video or text chat with a support person	6 (4.9)	
**Activity preferences (n=28 questions deployed)**			
	Show me my activity progress for the day	11 (39)	
	Remind me of my daily health intention	9 (32)	
	Provide easy exercise options nearby	9 (32)	
	Video demonstration of easy exercise	8 (29)	
	Something else^b^	5 (18)	
	Easy way to log my activity	4 (14)	

^a^Access to healthy preferred options or premade healthy options; a list of 3-5 quick and healthy meal options to prepare in advance; someone else to prepare healthy meals; inventory of food in my house or assistance figuring out healthy options without getting up; reduced focus on calories; suggest healthy options based on what I have in my house; motivational messaging and a dashboard of my progress; recipe suggestions; suggestions on healthy options in my area or just general food suggestions.

^b^Someone to go with me and hold me accountable; support me by checking in with me like a friend would.

### Usability, Acceptability, Feasibility, and Appropriateness

At the end of each study day, contextualized technology adaptation process questions regarding feasibility, acceptability, and appropriateness were deployed. The majority of respondents indicated that the number of daily prompts was “just right” (37/72, 51%), followed by “too low” (23/72, 32%), with “too high” (12/72, 17%) being the least frequently selected response. The majority of respondents viewed timing of prompts as “moderately helpful” (36/72, 50%), followed by “not at all helpful” (26/72, 36%), with the fewest responses indicating timing was “extremely helpful” (10/72, 14%). Responses to the final question, “What would have made this application more helpful,” shown in Table 2, indicated that users preferred personalized messaging and simplified self-monitoring options. Participants also had the option of adding free-text comments, as Table 3 shows.

**Table 2:** Pooled data for all participants (n=19, total possible responses n=69) responding to acceptability, feasibility, and appropriateness questions deployed at the end of each day.

What would have made this application more helpful?	Total responses, n (%)
Personalized text messages about how to reach my health goals	29 (42)
Provide an easy way to log my food and activity	28 (41)
Incentive for healthy choices (eg, collecting “points” to be used toward a reward)	28 (4
Anticipate sedentary behavior and send me activity suggestions	27 (39)
Anticipate unhealthy eating and send me healthier options nearby	25 (36)
Provide interactive video of exercise examples	23 (33)
Provide healthy meal options and recipes	23 (33)
Track and show me my activity throughout the day	21 (30)
Ability to video chat with a professional	5 (7)
Link to social media to share my progress with others	1 (1)
See a friend or loved one’s progress	0 (0)

**Table 3:** Postparticipation interview responses vs app-collected responses.

Question	Representative interview quote	Representative app quote
What did you like about the app?	I liked setting a health goal and then checking in at the end of the day.	I know how I should be feeling and behaviors I should be changing, but it is really hard when I am all alone to take the right steps...[the app] kept me busy so I wouldn’t get so sad.
What did you dislike about the app?	Too many prompts.	Hounding people to eat healthy and exercise is the #1 way to drive an emotionally fragile person away.
What would have made the app more useful?	Make logging easier.	Maybe a mood tracker would be helpful, and a visual of my progress.
What would make you more likely to continue using an app like this?	I want to be able to change the notification settings.	I’d like to be able to ask for help making eating choices when I’m ready to eat. Due to my disability, I don’t have a regular sleep schedule so I don’t eat meals at regular times.

In order to contrast results from traditional acceptability, feasibility, and appropriateness assessment methods with data collected via the app, we conducted semistructured interviews with 4 users representing high and low user engagement, contrasted with responses provided via free text through the app (Table 3). Follow-up interviewees in general gave positive feedback about the context, particularly about the intention setting at the beginning of the day, and anticipated or actual difficulty rating features. In contrast, feedback obtained through the app on a daily basis included more detail about the usability of the needs assessment tool, particularly with respect to what participants didn’t like. Respondents noted that their day-to-day schedules varied, in particular related to sleep and mood, so prescheduled “in-the-moment” needs assessment prompts weren’t always relevant and were perceived as annoying if they came at a time that was busy or stressful. Respondents also expressed a desire for the app to provide more emotional support, learn their patterns and behaviors, and personalize goals or substitute behaviors that consider a specific condition or disability.

## Discussion

### Principal Findings

We tested the utility of a novel mobile technology–based technique to capture both group-based and individually dynamic user needs and preferences. We conducted this assessment, which we term ecological daily needs assessment, alongside a basic mHealth intervention designed to support health behaviors linked to healthy weight management. There were 3 key findings. First, the technique demonstrated the ability to detect dynamic needs and preferences, which changed over time differentially within an individual, as well as between individuals. The technique collected group-level usability data suggesting specific adaptations for improving the app, as well as being informative for intervention adaptation. Second, the number of daily prompts was acceptable, but timing was rarely appropriate despite being during prespecified times indicated by the participant. Response rates declined significantly after 3 days, suggesting a possible threshold for collecting useful needs assessment data. Third, this approach to usability assessment yielded specific critical feedback, contrasted with less specific and generally positive or neutral feedback obtained from the more traditional method of semistructured interviews. These results highlight the importance of context in usability testing of mobile assessments, which can yield data relevant to dynamic needs and preferences at the group and individual levels.

Digital tools are commonly leveraged to temporally link active, passive, and metadata to improve user engagement. Despite this, mHealth apps are still disabled within weeks of download [38], suggesting that extensive digital phenotyping efforts are insufficient for optimizing user engagement [39,40]. Assessment of user preferences increases mHealth treatment engagement [41], but populations perceived as difficult to engage, such as individuals with psychiatric illnesses, are often excluded from treatment adaptation research, primarily due to concerns about the reliability of self-report measures due to cognitive limitations [42]. However, it is exactly these cognitive limitations that necessitate the need for usability and preference testing [41,43]. The extant research evaluating mHealth or digital weight loss treatments in young adults with mental illness is limited, but suggests that the unique needs and preferences of this population may have important implications for mHealth treatment development [44]. These results underscore the importance of ecologically valid data in developing interventions that effectively engage this population [45].

The needs assessment in this study was anchored within a behavior change framework based on existing treatments [29,46] to provide the user exposure to an intervention upon which they could consider their needs and preferences. In contrast, more traditional usability assessments conducted at the end of an intervention, even when paired with utilization data, are subject to recall bias and do not capture how end users experience the app in real time [47]. The complexity of programming needed to build a learning system from inception contributes to cost, and there are as yet few cost-benefit data to inform investment in artificial intelligence features like machine learning for health apps [48]. Developers of mHealth treatments in resource-constrained settings might more expeditiously begin app development with an anchored needs assessment in place to better understand end user needs, which could provide useful information regarding which data inputs are most relevant to employ in learning algorithms.

### Limitations

It is important to note limitations of this study. First, the number of participants was small, but within the recommended range for usability testing. Second, participation was limited to individuals owning iOS devices. Apps on non-iOS smart devices using the Android operating system, which are more commonly used in psychiatric populations [49], are needed. Third, although the needs assessment technique in this study prioritized participant-scheduled prompts, participants were not able to change the schedule or initiate a response when they encountered a need outside of the schedule, resulting in potential missed opportunities to capture more nuanced aspects of individual variability in needs and preferences. Fourth, important questions remain regarding the validity and relevance of our observations for mHealth treatment development. If individually dynamic needs and preferences are relevant, what is the best way to accurately capture this information? And how can this information be meaningfully incorporated into behavioral mHealth intervention development and adaptation? Empirical testing is needed to determine whether capturing individually dynamic data leads to superior mHealth interventions and outcomes.

### Conclusions

The results of this study provide insights that may inform the development of self-adapting treatments, which this study and others have identified as particularly germane to engagement of individuals with chronic health concerns, including mental health conditions [27,50]. In populations where app engagement is linked to treatment outcome, or in settings where funding for development is limited, early usability testing of digital features, using the digital context, is a low-cost option for determining which aspects of development to prioritize with limited funding. The resulting contextually relevant information might be particularly useful in guiding real-time treatment adaptation while limiting in-person contact, which will likely be important for the future of clinical research in vulnerable populations during public health events like the coronavirus pandemic [51]. Additional study is needed to determine whether a mobile needs assessment can usefully inform behavioral treatment development for diverse patient populations and operating systems.

## Appendix

Multimedia Appendix 1 Supplementary materials.

## Abbreviations

mHealth: mobile health

## References

[1] Buttorff C, Ruder T, Bauman M (2017). Multiple Chronic Conditions in the United States.

[2] (2020). NHE Fact Sheet.

[3] Krebs P, Duncan DT (2015). Health app use among US mobile phone owners: a national survey. JMIR Mhealth Uhealth.

[4] Yardley L, Morrison L, Bradbury K, Muller I (2015). The person-based approach to intervention development: application to digital health-related behavior change interventions. J Med Internet Res.

[5] Bowling A, Ebrahim S (2001). Measuring patients' preferences for treatment and perceptions of risk. Qual Health Care.

[6] Franz MJ, VanWormer JJ, Crain AL, Boucher JL, Histon T, Caplan W, Bowman JD, Pronk NP (2007). Weight-loss outcomes: a systematic review and meta-analysis of weight-loss clinical trials with a minimum 1-year follow-up. J Am Diet Assoc.

[7] Dombrowski SU, Knittle K, Avenell A, Araújo-Soares V, Sniehotta FF (2014). Long term maintenance of weight loss with non-surgical interventions in obese adults: systematic review and meta-analyses of randomised controlled trials. BMJ.

[8] Byrne S, Cooper Z, Fairburn C (2003). Weight maintenance and relapse in obesity: a qualitative study. Int J Obes Relat Metab Disord.

[9] Yu Z, Sealey-Potts C, Rodriguez J (2015). Dietary self-monitoring in weight management: current evidence on efficacy and adherence. J Acad Nutr Diet.

[10] Johnston CA, Tyler C, Foreyt JP (2007). Behavioral management of obesity. Curr Atheroscler Rep.

[11] Melamed OC, Fernando I, Soklaridis S, Hahn MK, LeMessurier KW, Taylor VH (2019). Understanding engagement with a physical health service: a qualitative study of patients with severe mental illness. Can J Psychiatry.

[12] Firth J, Rosenbaum S, Stubbs B, Gorczynski P, Yung AR, Vancampfort D (2016). Motivating factors and barriers towards exercise in severe mental illness: a systematic review and meta-analysis. Psychol Med.

[13] Nicol G, Worsham E, Haire-Joshu D, Duncan A, Schweiger J, Yingling M, Lenze E (2016). Getting to more effective weight management in antipsychotic-treated youth: a survey of barriers and preferences. Child Obes.

[14] Lenhart A (2015). https://www.pewresearch.org/internet/2015/04/09/teens-social-media-technology-2015/.

[15] Jacobs S, Radnitz C, Hildebrandt T (2017). Adherence as a predictor of weight loss in a commonly used smartphone application. Obes Res Clin Pract.

[16] Patrick K, Griswold WG, Raab F, Intille SS (2008). Health and the mobile phone. Am J Prev Med.

[17] De Hert M, Correll CU, Bobes J, Cetkovich-Bakmas M, Cohen D, Asai I, Detraux J, Gautam S, Möller H, Ndetei DM, Newcomer JW, Uwakwe R, Leucht S (2011). Physical illness in patients with severe mental disorders. I. Prevalence, impact of medications and disparities in health care. World Psychiatry.

[18] Wiltsey Stirman S, Baumann AA, Miller CJ (2019). The FRAME: an expanded framework for reporting adaptations and modifications to evidence-based interventions. Implement Sci.

[19] Schnall R, Rojas M, Bakken S, Brown W, Carballo-Dieguez A, Carry M, Gelaude D, Mosley JP, Travers J (2016). A user-centered model for designing consumer mobile health (mHealth) applications (apps). J Biomed Inform.

[20] McCurdie T, Taneva S, Casselman M, Yeung M, McDaniel C, Ho W, Cafazzo J (2012). mHealth consumer apps: the case for user-centered design. Biomed Instrum Technol.

[21] Shiffman S, Stone AA, Hufford MR (2008). Ecological momentary assessment. Annu Rev Clin Psychol.

[22] Ben-Zeev D, Brenner CJ, Begale M, Duffecy J, Mohr DC, Mueser KT (2014). Feasibility, acceptability, and preliminary efficacy of a smartphone intervention for schizophrenia. Schizophr Bull.

[23] Huang Y, Benford S, Blake H (2019). Digital interventions to reduce sedentary behaviors of office workers: scoping review. J Med Internet Res.

[24] Fisher AJ (2015). Toward a dynamic model of psychological assessment: implications for personalized care. J Consult Clin Psychol.

[25] Moore RC, Depp CA, Wetherell JL, Lenze EJ (2016). Ecological momentary assessment versus standard assessment instruments for measuring mindfulness, depressed mood, and anxiety among older adults. J Psychiatr Res.

[26] Floch J, Zettl A, Fricke L, Weisser T, Grut L, Vilarinho T, Stav E, Ascolese A, Schauber C (2018). User needs in the development of a health app ecosystem for self-management of cystic fibrosis: user-centered development approach. JMIR Mhealth Uhealth.

[27] Setiawan IMA, Zhou L, Alfikri Z, Saptono A, Fairman AD, Dicianno BE, Parmanto B (2019). An adaptive mobile health system to support self-management for persons with chronic conditions and disabilities: usability and feasibility studies. JMIR Form Res.

[28] Hardeman W, Houghton J, Lane K, Jones A, Naughton F (2019). A systematic review of just-in-time adaptive interventions (JITAIs) to promote physical activity. Int J Behav Nutr Phys Act.

[29] Tabak RG, Strickland JR, Stein RI, Dart H, Colditz GA, Kirk B, Dale AM, Evanoff BA (2018). Development of a scalable weight loss intervention for low-income workers through adaptation of interactive obesity treatment approach (iOTA). BMC Public Health.

[30] Stein RI, Strickland JR, Tabak RG, Dale AM, Colditz GA, Evanoff BA (2019). Design of a randomized trial testing a multi-level weight-control intervention to reduce obesity and related health conditions in low-income workers. Contemp Clin Trials.

[31] Degroote L, DeSmet A, De Bourdeaudhuij I, Van Dyck D, Crombez G (2020). Content validity and methodological considerations in ecological momentary assessment studies on physical activity and sedentary behaviour: a systematic review. Int J Behav Nutr Phys Act.

[32] Bandura A (1977). Self-efficacy: toward a unifying theory of behavioral change. Psychol Rev.

[33] Sweller J (1994). Cognitive load theory, learning difficulty, and instructional design. Learn Instruct.

[34] Ahmad FA, Payne PRO, Lackey I, Komeshak R, Kenney K, Magnusen B, Metts C, Bailey T (2020). Using REDCap and Apple ResearchKit to integrate patient questionnaires and clinical decision support into the electronic health record to improve sexually transmitted infection testing in the emergency department. J Am Med Inform Assoc.

[35] Lyon AR, Wasse JK, Ludwig K, Zachry M, Bruns EJ, Unützer J, McCauley E (2016). The contextualized technology adaptation process (CTAP): optimizing health information technology to improve mental health systems. Adm Policy Ment Health.

[36] Nielsen J, Landauer TK (1993). A mathematical model of the finding of usability problems.

[37] Macefield R (2009). How to specify the participant group size for usability studies: a practitioner's guide. J Usabil Stud.

[38] Dorsey ER, Yvonne CY, McConnell MV, Shaw SY, Trister AD, Friend SH (2017). The use of smartphones for health research. Acad Med.

[39] Jain SH, Powers BW, Hawkins JB, Brownstein JS (2015). The digital phenotype. Nat Biotechnol.

[40] Insel TR (2017). Digital phenotyping: technology for a new science of behavior. JAMA.

[41] Søgaard Neilsen A, Wilson RL (2019). Combining e-mental health intervention development with human computer interaction (HCI) design to enhance technology-facilitated recovery for people with depression and/or anxiety conditions: an integrative literature review. Int J Ment Health Nurs.

[42] Morrison LG, Yardley L, Powell J, Michie S (2012). What design features are used in effective e-health interventions? A review using techniques from Critical Interpretive Synthesis. Telemed J E Health.

[43] Archer N, Keshavjee K, Demers C, Lee R (2014). Online self-management interventions for chronically ill patients: cognitive impairment and technology issues. Int J Med Inform.

[44] Aschbrenner KA, Naslund JA, Shevenell M, Mueser KT, Bartels SJ (2016). Feasibility of behavioral weight loss treatment enhanced with peer support and mobile health technology for individuals with serious mental illness. Psychiatr Q.

[45] Naslund JA, Aschbrenner KA (2019). Digital technology for health promotion: opportunities to address excess mortality in persons living with severe mental disorders. Evid Based Ment Health.

[46] Nicol GE, Kolko R, Lenze EJ, Yingling MD, Miller JP, Ricchio AR, Schweiger JA, Findling RL, Wilfley D, Newcomer JW (2019). Adiposity, hepatic triglyceride, and carotid intima media thickness during behavioral weight loss treatment in antipsychotic-treated youth: a randomized pilot study. J Child Adolesc Psychopharmacol.

[47] Chen AT, Wu S, Tomasino KN, Lattie EG, Mohr DC (2019). A multi-faceted approach to characterizing user behavior and experience in a digital mental health intervention. J Biomed Inform.

[48] Wolff J, Pauling J, Keck A, Baumbach J (2020). The economic impact of artificial intelligence in health care: systematic review. J Med Internet Res.

[49] Young AS, Cohen AN, Niv N, Nowlin-Finch N, Oberman RS, Olmos-Ochoa TT, Goldberg RW, Whelan F (2020). Mobile phone and smartphone use by people with serious mental illness. Psychiatr Serv.

[50] Thornton LK, Kay-Lambkin FJ (2018). Specific features of current and emerging mobile health apps: user views among people with and without mental health problems. Mhealth.

[51] Padala PR, Jendro AM, Padala KP (2020). Conducting clinical research during the COVID-19 pandemic: investigator and participant perspectives. JMIR Public Health Surveill.

