# Supplementary material for: A Smartphone-Based Technique to Detect Dynamic User Preferences for Tailoring Behavioral Interventions: Observational Utility Study of Ecological Daily Needs Assessment
Source: JMIR Mhealth Uhealth. 2020 Nov 13;8(11):e18609. doi: 10.2196/18609 (PMC7695533; doi:10.2196/18609)
Supplement: Multimedia Appendix 1 [file mhealth_v8i11e18609_app1.docx]

**Ecological Daily Needs Assessment: usability testing of a smartphone-based technique to detect dynamic user preferences for tailoring behavioral interventions**

**Multimedia Appendix Table of Contents**

Appendix 1: Study Protocol pages 2-4

Appendix 2: Additional study methodology and figures pages 5-6

References page 6

Appendix 1

**Study Title**

An Ecological Daily Needs Assessment (EDNA) to develop a Mobile Health Behavior Change Application for Individuals with Psychiatric Disorders

**Study Aims**

The purpose of this project is to conduct a needs assessment for behavioral weight loss interventions in individuals with psychiatric disorders. This project extends results from previous studies indicating that individuals with psychiatric illnesses have different needs, preferences for and barriers to making health behavior changes than the general population. We aim to collect data regarding eating behaviors, previous attempts at weight loss, and openness to mobile health programming to make health behavior changes. The results of this study will impact future behavioral weight loss interventions for individuals with mental illness. The data collected will be informative for personalized and effective behavioral methods of mobile health technology to target lifestyle habits of people with mental illness. Participants will download the study application in Apple ResearchKit, and will be asked to complete study questionnaires and tasks on their smartphone or other web-enabled iOS mobile device. The questionnaires will consist of one demographics questionnaire, one preference for health behavior prompts questionnaire, and one week of the daily needs assessment that will send messages and prompts to participants based on the times of day that they said they preferred to be prompted. At the end of the one week needs assessment, participants will be asked to complete a short end of study questionnaire.

- We propose to conduct a needs assessment with the purpose of developing an effective mobile behavioral weight loss (BWL) intervention for individuals with severe mental illness (SMI).
- We will use Ecological Daily Needs Assessment (EDNA), in which the mobile technology collects needs assessment data in real time. An ecological, real-time needs assessment design can provide more accurate information on the needs of individuals with mental illness and the health challenges that they face to live longer, healthier lives.

**Background and Significance**

Individuals with psychiatric conditions are at greater risk than the general population for overweight and obesity, leading to higher rates of diabetes and death from cardiovascular disease.[1] Some of this risk is attributable to psychotropic medications that cause weight gain; additional risk is attributable to environment, lifestyle and disparity in access to primary care.[2] However, it has been demonstrated that individuals, including youth, with mental illness can benefit from behavioral interventions to increase health behaviors, lose weight and improve metabolic profile.[3,4] Unfortunately, these programs are difficult to implement as they are costly and lengthy, leading to high dropout rates and poor outcomes when attempts are made to disseminate them widely.[5] Our research has suggested that youth and their parents are open to and would be more likely to participate in behavioral weight loss treatment if it included technology.[6 The purpose of this project is to obtain more detailed information about the needs and preferences of individuals with psychiatric conditions, and their parents for youth younger than 18 years old, for behavioral weight loss treatment.

**Recruitment Methods**

- Ads/Brochures/Posters/News Release/Fliers
- Email or letters
- Website or Social Media
- Other Materials - The study will be listed in iTunes under Apple Researchkit for users to download and learn more about.
- Existing Registry/database
- Another Registry/database
  - - Washington University Volunteers for Health research participant registry is a local registry that matches local volunteers to studies at Washington University.
    - Research Match database (www.ResearchMatch.com) is a national registry that matches participants to studies that we will use for recruitment.

**Enrollment & Consent Procedures**

Potential participants will be identified by our typical recruitment procedures, which include placement of posters, flyers and brochures in the appropriate clinical settings. The PI will also utilize social media to educate the public about the study and provide information on how to enroll or who to contact with questions - the social media platforms for recruitment will be Facebook and Twitter. Finally, the PI will also post information about the study on the laboratory website: healthymind.wustl.edu. The study will be part of Apple ResearchKit, so it will be advertised in the Apple App store for download through iTunes. The study team will make every effort to minimize any sense of coercion or undue influence during the consent process. Specifically, the consent process will include multiple messages about being able to ask questions at any point, being able to stop the process at any point, and will be reassured that their treatment will in no way be affected by participation or non-participation in the study.

If a participant is interested, he/she will need to be an Apple iPhone user and learn more about the study by searching the Apple App store for ResearchKit study apps or by name. If a potential participant has any questions about the study, study contact information (email address and phone number) will be included in the advertisements, in Apple ResearchKit and in the consent. Interested participants will have to search the study in ResearchKit in the App store and download it to begin the process of learning more about the study and answering the eligibility questions.

If the participant is still interested, eligibility questions will be launched. If the participant is eligible, he/she will be given an option to begin the consent process and view the full consent. Apple ResearchKit has a standardized consent platform that allows all of the sections of the consent to be addressed while the participant swipes and is given the option to "learn more" about each section. Due to Apple's standardized consent platform, the consent screens are limited to a few lines of text, so the participant will be required to view the "learn more" text to see the full explanation of each section of consent before advancing to the next section. The participant always has the option of downloading the full consent form for review and going back to review any prior sections. After going through all of the sections of the mobile consent and reviewing the full consent form in its entirety, the participant will be asked to provide a signature for informed consent and will have the option to enter an email address so that a pdf copy of the ICF can be emailed to them for their records.

Examples of what the process of downloading the app looks like can be found in Appendix 2. Apple ResearchKit requires a written privacy policy that participants can easily locate (both, during the consent process and anytime on the study website) if they have any questions or concerns about their information and data collected.

All study procedures will be completed through the mobile application on the participant's smart device. After downloading the application through Apple ResearchKit in the App store, the participant will see a welcome screen and will respond to eligibility questions. If eligible the mobile consent process will be launched. After informed consent, the study application will launch the preferences for health prompts questionnaire to ask participants what times of day they prefer to be sent messages about their health behaviors. This will take about 5-10 minutes to complete. Then, the demographics questionnaire will be launched. This will also take about 5-10 minutes to complete. The next day will be the start of the 14 day needs assessment program; the participant will begin to receive messages based on the times of the day they told us that they would like to be sent messages about their health behaviors and questions about then they are about to eat. At the end of each day, participants will complete a short questionnaire about how they felt about the program. At the end of the day on day 14 of the needs assessment, participants will be asked to complete a satisfaction questionnaire about the program. Participants always have the option of declining to respond to the messages or answering any questions in the questionnaires.

**Participants**

Participants will be contributing important information to the general knowledge about unique needs and preferences of individuals with psychiatric conditions to change or improve their health behaviors. Participants will be able to participate in a simple "trial" experience of using a mobile application to help them change health behaviors, and their feedback will be used to develop a more effective intervention for others that have conditions and lifestyle challenges similar to theirs.

Society will benefit from this study, as it aims to collect ecologically valid data regarding the unique needs and preferences of this population. Individuals with mental illnesses are at increased risk for obesity, diabetes, cardiovascular disease and early mortality from these conditions. Effective health behavior change treatments are critically needed to decrease the disease burden in this population and allow them to live longer, healthier and more productive lives. Additionally, interventions that decrease the burden of these conditions in mentally ill individuals will lead to lower health care costs for this population.

Children/minors will be given only a verbal description of the study information and asked to assent verbally - Minor participants will be present when the consent document is read and their parent/legal guardian assent consents to the study over the phone and minor participants will provide verbal assent.

Although it is possible for a minor to turn 18 years of age during the 2 weeks of study participation, we will not be re-consenting them as adult participants because the study procedures on the mobile application will be interrupted during the 2 week needs assessment. Study procedures will only last one week and it is important that the 7 days of needs assessment prompts and questionnaires are answered consecutively. Contacting the participant and obtaining consent may delay data collection and programming. In addition, the study is minimal risk and participants may choose not to answer or withdraw at any time.

Participants completing the 2-week study will be entered into a monthly drawing to receive a $50 VISA gift card.

*Inclusion Criteria*

- at least 18 years old (no upper age limit)
- have been diagnosed with a psychiatric disorder
- own an iOS mobile device
- willing to participate in a 2-week study
- willing and able to keep their device with them to study prompts for most of the day for 2 weeks

*Exclusion Criteria*

- Prior diagnosis of anorexia nervosa, bulimia, binge eating disorder or other eating disorders.

**Power and Analytic Approach:**

Since the purpose of this study is to characterize the population and collect information on the needs and preferences of the population for the development of treatment interventions, no power analysis is needed. In order to obtain enough responses to be representative of the population under study, we estimate that 35 participants are needed for feedback and pilot data.

**Results:**

- Time to app discontinuation
- Overall response rate during the study period
- Descriptive characteristics of participant responses to needs assessment prompts
- Frequency of response type to specific needs and preferences questions
- Qualitative feedback regarding participant experience using the app collected via free-text prompts through the app and semi-structured interviews with participants exhibiting high (>80%) and low (<20%) response rates during the study period

Appendix 2

***Additional Description of Mobile Application Development***

Initial versions of the application (v1.6.3 - v1.6.7) were for minor bug fixes and to iteratively develop and test the electronic signed consent process, which was completed in August 2017. Recruitment took place from September 2017 to March 2018, with one final minor bug fix to v 1.7.4 in February 2018.


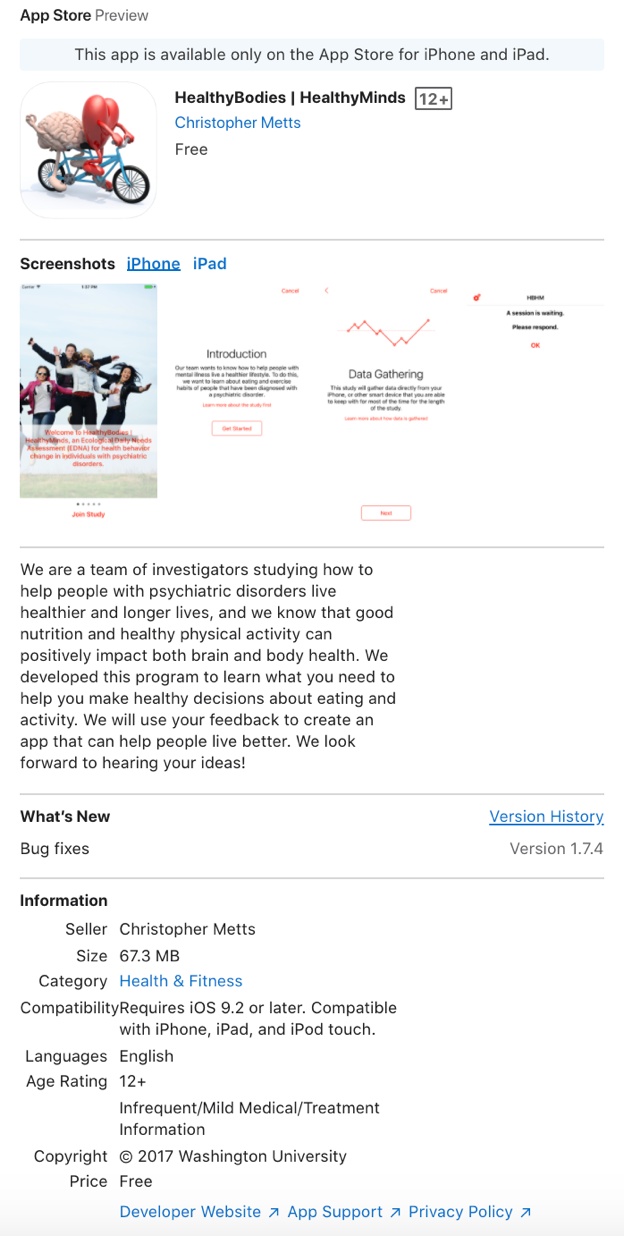

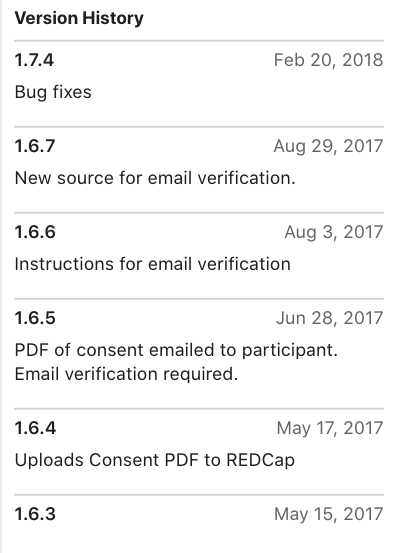

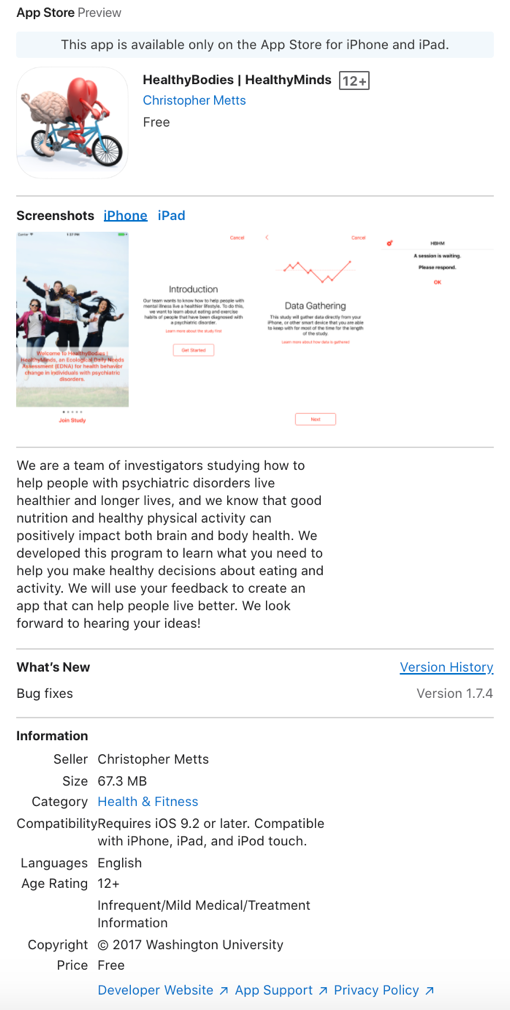


In the App Store Preview, potential participants could view the initial welcome screen, screening questions, and basic information about the application. The only place in the preview or application where Washington University is mentioned is mentioned is in the copywrite information.

***Additional Detail Regarding Recruitment Procedures***

A multimedia FaceBook advertisement (Figure 1) included a 10-second animation of the application, which briefly described the purpose of the study, showing a user swiping through consent pages and engaging with health intention setting and needs assessment screens (Figures 2a and 2b). Potential participants could link directly to the app store to download the application and begin screening and consent processes.

**Figure 2a: Health Intention Setting**


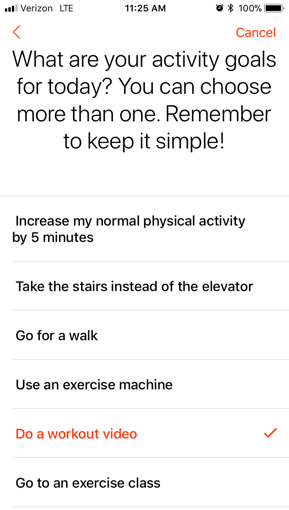

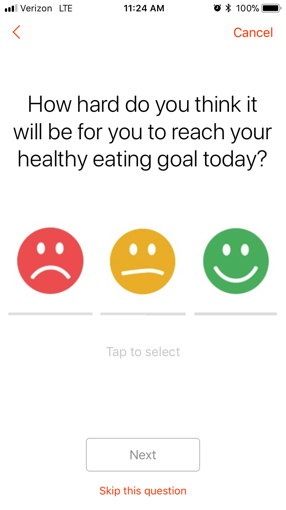


**Figure 2b: Needs Assessment**


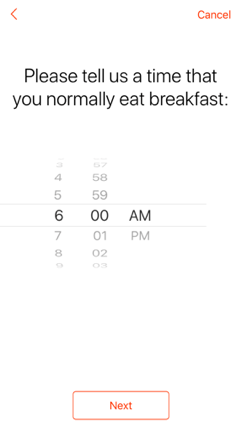

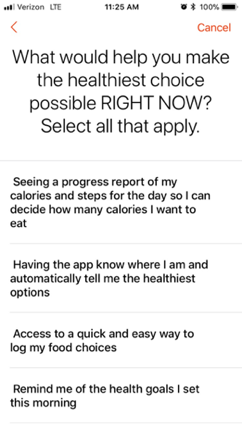

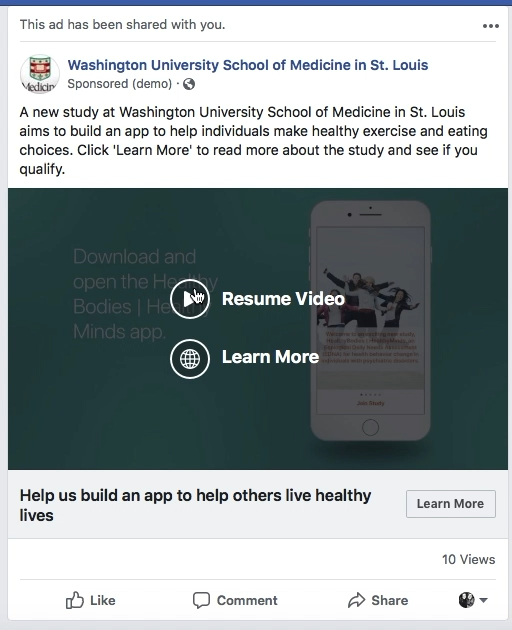


**Figure 1: FaceBook Multimedia Recruitment Ad**

***Additional Detail Regarding Assessments and Data Management***

**Healthy eating goal options:** a) eating out less, b) drink less regular soda c) limit sugar intake d) drink more water e) order grilled instead of fried food when I eat out f) choose lower fat options g) eat more vegetables or h) other (enter free text).

**Healthy activity goal options:** a) increase my normal activity by 5 minutes b) take the stairs instead of the elevator c) go for a walk d) use an exercise machine e) spend less time sitting f) do a workout video g) go to an exercise class or h) other (entered via free text).

**Semi structured Interviews with High- (>80% response rate) and Low-engagement Users (<25% response rate):** 1) What did you like about the application? 2) What did you dislike about the application? 3) What would have made the application more useful? And 4) What would make you more likely ro continue using an application like this?

**Data Management:** Study data were collected and managed using REDCap electronic data capture tools[7] hosted at Washington University. Through the RedCap user-interface, investigators can create HIPAA compliant participant surveys and push-notification event schedules that are fully integrated with the supporting research databases. Status/Post implements branching-logic used in defining REDCap data collection instruments or surveys, with additional features including hidden fields, randomization of question order, and branching-logic based on variables held on the device. The usability test was designed as a fully remote, interactive experience that could be completed on a mobile device from start to finish, including digital screening and consent. At initialization and following queries to the application program interface (API), data was pushed to REDCap as collected from the user-interface, with caching of data when a network connection was not available.

**References**

1. Lyon AR, Wasse JK, Ludwig K, Zachry M, Bruns EJ, Unutzer J, McCauley E. The Contextualized Technology Adaptation Process (CTAP): Optimizing health information technology to improve mental health systems. Adm Policy Ment Health. 2016;43(3):394-409. PMC4536193.
2. Colton CW, Manderscheid RW. Congruencies in increased mortality rates, years of potential life lost, and causes of death among public mental health clients in eight states. Prev Chronic Dis. 2006;3(2):A42. PMC1563985.
3. Walker ER, McGee RE, Druss BG. Mortality in mental disorders and global disease burden implications: a systematic review and meta-analysis. JAMA Psychiatry. 2015;72(4):334-341. PMC4461039.
4. Morrato EH, Campagna EJ, Brewer SE, Dickinson LM, Thomas DS, Miller BF, Dearing J, Druss BG, Lindrooth RC. Metabolic testing for adults in a state medicaid program receiving antipsychotics: remaining barriers to achieving population health prevention goals. JAMA Psychiatry. 2016;73(7):721-730.
5. Morrato EH, Brewer SE, Campagna EJ, Dickinson LM, Thomas DS, Druss BG, Miller BF, Newcomer JW, Lindrooth RC. Glucose testing for Adults receiving medicaid and antipsychotics: a population-based prescriber survey on behaviors, attitudes, and barriers. Psychiatr Serv. 2016;67(7):798-802. PMC4939591.
6. Knowler WC, Fowler SE, Hamman RF, Christophi CA, Hoffman HJ, Brenneman AT, Brown-Friday JO, Goldberg R, Venditti E, Nathan DM. 10-year follow-up of diabetes incidence and weight loss in the Diabetes Prevention Program Outcomes Study. Lancet. 2009;374(9702):1677-1686. PMC3135022.

1. Harris PA, Taylor R, Thielke R, Payne J, Gonzalez N, Conde JG. Research electronic data capture (REDCap)--a metadata-driven methodology and workflow process for providing translational research informatics support. J Biomed Inform. 2009;42(2):377-81.PMC2700030
